# Supplementary material for: Effect of the Soil Matric Potential on the Germination Capacity of Prosopis chilensis, Quillaja saponaria and Cryptocarya alba from Contrasting Geographical Origins
Source: Plants (Basel). 2022 Nov 2;11(21):2963. doi: 10.3390/plants11212963 (PMC9656835; doi:10.3390/plants11212963)

Table S1. Defoliation or damage classes to define the Health Status of trees.

Class	Defoliation (%)	Definition
0	0–10	Trees without damage/no discoloration
1	11–25	Slightly damaged/light discoloration
2	26–60	Moderately damaged/moderate discoloration
3	61–90	Severely damaged/severe discoloration
4	91–100	Dry or dead tree

[48]

Table S2. Martonne arid index

Value	Classification
>40	wet
30–40	subhumid
20–30	semiarid
10–20	arid
5–10	subdesert
<5	desert

its value is calculated using the formula $I=P/(T+10)$ from the data obtained from the climograms (where T is the average annual temperature and P is the total annual quantity of water that falls in litres). According to this index, each geographical place will be classified according to its degree of aridity.

Figure S1. Water retention characteristic curve of sustrate.

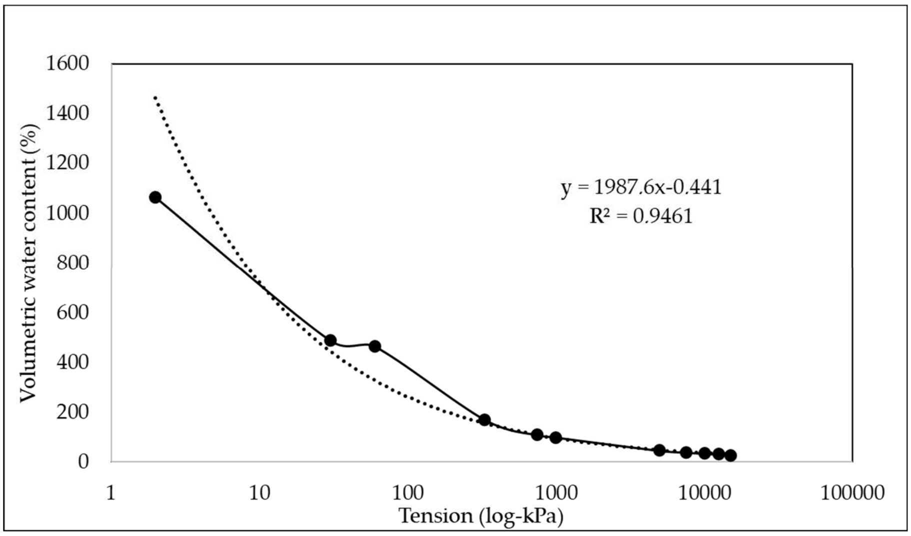

Supplement: Supplementary file 1 [file plants-11-02963-s001.zip › plants-1954225-supplementary.pdf]
